# Full bladder, empty rectum? Revisiting a paradigm in the era of adaptive radiotherapy

**DOI:** 10.1007/s00066-024-02306-7

**Published:** 2024-10-29

**Authors:** Hanna Malygina, Hendrik Auerbach, Frank Nuesken, Jan Palm, Markus Hecht, Yvonne Dzierma

**Affiliations:** Department of Radiotherapy and Radiation Oncology, Saarland University Medical Centre, Kirrberger Str. 100, 66421 Homburg, Saar Germany

**Keywords:** Prostate cancer, Online adaptive radiotherapy, Rectum volume, Bladder volume, Varian Ethos

## Abstract

**Background and Purpose:**

Many patients find it challenging to comply with instructions regarding rectum and bladder filling during pelvic radiotherapy. With the implementation of online adaptive radiotherapy, the reproducibility of organ volumes is no longer a prerequisite. This study aims to analyze the sparing of the bladder and the posterior rectum wall (PRW) in conditions of full versus empty bladder and rectum.

**Methods:**

280 fractions from 14 patients with prostate cancer who underwent adaptive radiotherapy using the Varian Ethos system were analyzed post-hoc. Various metrics for the bladder and PRW were correlated with respect to organ volume.

**Results:**

Our analysis quantitatively confirms the advantage of a full bladder during radiotherapy, as metrics V48Gy and V40Gy significantly inversely correlate with bladder filling for each patient individually. While bladder volume did not show a gradual decrease over the course of radiotherapy, it was observed to be higher during planning CT scans compared to treatment sessions. A full rectum condition either significantly improved (in 2 out of 7 patients) or at least did not impair (in 5 out of 7 patients) PRW sparing, as represented by the V30Gy metric, when patients were compared individually. The average V30Gy across all patients demonstrated a significant improvement in PRW sparing for the full rectum condition, with a $p$-value of 0.039.

**Conclusion:**

Despite the implementation of adaptive therapy, maintaining a high bladder filling remains important. However, the recommendation for rectum filling can be abandoned, as reproducibility is not critical for adaptive radiotherapy and no dosimetric advantage per se is associated with an empty rectum. Patients may even be encouraged not to void their bowels shortly before treatment, as long as this is tolerated over the treatment session.

The online version of this article (10.1007/s00066-024-02306-7) contains supplementary material, which is available to authorized users.

## Introduction

It has been an established paradigm in prostate radiotherapy to recommend a full bladder and empty rectum [1–3]. While bladder filling directly and obviously improves bladder dose sparing by causing large parts of the organ to expand and move away from the planning target volume and hence outside the high dose region [1, 4, 5], the rationale for voiding the rectum is less intuitive, arising mainly from the need for reproducibility. In fact, some institutions use endorectal balloons or similar devices to artificially distance the posterior rectal wall from the target volume [6–9]. For patient comfort, however, this is often omitted, and an empty rectum is then recommended to ascertain best reproducibility of the anatomy in every fraction. In the era of online adaptive radiotherapy, reproducibility is no longer an essential prerequisite [10]. On the contrary, rectal filling might contribute to distancing the posterior rectal wall (PRW) from the prostate and hence high dose regions. Even if a consistently full rectum is not possible to achieve over the full course of a radiotherapy series, random variations may still offer a dosimetric improvement over a recommended empty scenario.

Given that it is clinically challenging for many patients to comply with both rectum and bladder filling instructions, adaptive radiotherapy may thus warrant relaxing the recommendation of rectum filling. Patients would then only be asked to adhere to a standardized drinking protocol, which would be considered less stressful. Additionally, this may facilitate the clinical workflow since situations in which the patient would be taken off the treatment table after positioning and cone-beam computed tomography (CBCT), asked to void their bowel, and return for another treatment attempt, would be avoided. This would not only prevent additional strain and exhaustion to the patient, but it would also save time in a busy clinical schedule, furthermore reducing the need for repeated CBCT-verification and the associated imaging dose.

In the study, we included the subgroup of 14 patients with low and intermediate risk localized prostate cancer who had moderate or high risk of seminal vesicle involvement according to the criteria specified in the CHHiP trial [11] and were treated at the Varian Ethos system at our institution. The doses to the bladder and posterior rectal wall are evaluated as a function of the daily organ volume as determined on the kV-CBCT. This represents a realistic data set of the actual organ volumes as they are observed over a treatment course with standardized drinking and rectum voiding instructions.

To our knowledge, this topic has not been addressed for daily adaptive treatment in the recent literature so far. We therefore believe the results will be interesting for the clinical management of adaptive pelvic radiotherapy in the realistic clinical setting. Furthermore, we hope this will provide an incentive to critically re-evaluate which paradigms from “classical” image-guided radiotherapy still hold in the era of daily adaptive radiotherapy.

## Patients and methods

### Adaptive Radiotherapy

kV-CBCT-based adaptive radiotherapy with the Varian Ethos system (Varian, Palo Alto, California) [12, 13] is planned and performed in a dedicated treatment planning, delivery and review system with a pre-defined workflow. On the basis of the planning CT, the treatment intent is specified, including dose prescription, planning objectives, and organs at risk (OAR) to be delineated. Based on the defined treatment setting (in this case, prostate), particular contours are defined as “influencers”, e.g. the bladder. It is assumed that changes in these contours will influence the shape of the clinical target volume (CTV) and planning target volume (PTV) in the adaptation process. A treatment plan is then created on the basis of the planning CT, choosing from a small number of possible geometries (intensity modulated radiotherapy (IMRT) with 7, 9, or 12 equidistant beams or volumetric modulated arc therapy (VMAT) with 2 arcs in the case of a non-lateralized target). Once the plan is accepted, it is available for treatment. When the patient comes for treatment, positioning will be performed and a kV-CBCT will be acquired. After image quality checking by the user, the contours will be automatically propagated to the kV-CBCT of the day, where they must be revised and can be changed by the user. The kV-CBCT will then be used to calculate two dose distributions (actually, these will be calculated on the planning CT after deformable registration to the kV-CBCT to ensure Hounsfield unit accuracy): the dose distribution from the original plan as it would result if applied on the changed anatomy, and a newly-reoptimized plan with the original intent and plan parameters, optimized on the anatomy of the day. The user can then compare these two plans and decide which one to accept for treatment. In general, the adapted plan offers improved dosimetric quality, and in our institution it is almost always selected for treatment.

### Patient Selection and Treatment Characteristics

Since the introduction of daily adaptive radiotherapy with the Varian Ethos system at our institution in July 2023, 37 patients (856 fractions) had been treated by the beginning of our study, most of these with pelvic radiotherapy indications, mainly prostate cancer. Patients with primary prostate cancer radiotherapy are treated at our institution with an in-house protocol based on the CHHiP trial [11]. In this post-hoc study, we included all 14 patients who has moderate or high risk of seminal vesicles involvement that is defined as in [11]. The patient were treated before the study and the analysis approach were specified (thus it is an exploratory experimental analysis). The CTV includes the prostate and the proximal 2 cm of seminal vesicles as well as an isotropic expansion. The CTV to PTV margins is isotropic and accounts for the uncertainties of the planning and the anatomy. The dose prescription is in the form of a nested simultaneously integrated boost (SIB), such that PTV/SIB1/SIB2 receive 48/57.6/60 Gy in 20 week-daily fractions of 2.4/2.88/3 Gy per fraction. Our in-house dose objectives on the organs at risk (OAR’s) are based on the CHHiP [11], PROFIT [14], PACE-B [15] and PACE-C [16] trials, and those for the organs considered in this study are listed in Table 1.

**Table 1:** In-house clinically relevant dose constrains for considered organs at risk. Optimal and alternative objectives for each dose and each organ. The constrain marked with * is not clinically relevant and is used only for the plan optimisation

Organ at risk	Dose,	Volumes	
	Gy	Optimal	Alternative
Rectum	24	$<70\%$	–
	32	$<51\%$	–
	40	$<38\%$	$<50\%$
	48	$<27\%$	$<35\%$
	52	$<22\%$	$<30\%$
	56	$<15\%$	$<25\%$
	60	$<1\%$	$<3\%$
Bladder	40	$<50\%$	–
	48	$<25\%$	–
	60	$<5\%$	–
Posterior	37	$<5\%$	–
Rectal	42	$0\,\mathrm{cm}^{3}$	–
Wall	30*	$<5\%$	–

Only those patients were included for whom no session was interrupted, as this is differently logged by the system and precludes evaluation of the organ volume. Patients were aged between 65 and 83 years, the prostate volume ranged between 44 and 161 $\mathrm{cm}^{3}$ (average: 72 $\mathrm{cm}^{3}$) (Table 2). All except one patient were treated at the Varian Ethos system using intensity-modulated radiotherapy (IMRT) with 7, 9, or 12 beams using 6 MV flattening-filter-free photons, one patient received two sessions of volume-modulated arc therapy (VMAT) with two arcs and 18 sessions of IMRT (the change was made because time for adaptation of the VMAT plan was deemed too long). All patients gave written informed consent for retrospective, pseudonymized analysis of their data in the context of the PRAIRIE trial protocol (Ethics vote 111/23 by Ärztekammer des Saarlandes).

**Table 2:** Demographic characteristics including age, cancer stage, Gleason grade, the latest PSA value before or shortly after the start of the treatment, as well as the plan modality

Age, years	Mean	min–max
	72.5	62–83
Cancer stage	# of patients	% of patients
T1b	1	7.1
T1c	7	50
T2a	1	7.1
T2b	0	0
T2c	5	35.7
Gleason grade	# of patients	% of patients
7	2	14.3
7a	7	50
7b	3	21.4
8	2	14.3
PSA, ng/ml	Mean	min–max
	6.6	0.08–17.9
Plan	# of fractions	% of fractions
IMRT 07	6	2.1
IMRT 09	66	23.6
IMRT 12	206	73.6
ARC 2	2	0.7

### Statistical Analysis

**Fig. 1:**
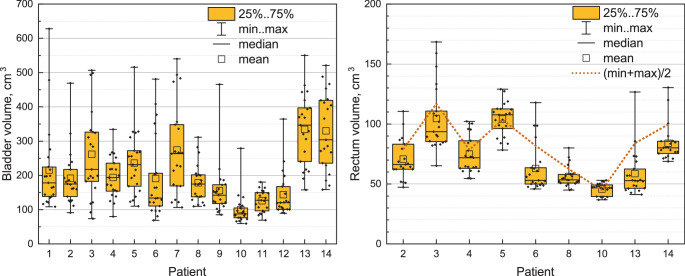
Boxplots for the distributions of patients’ bladder **(a)** and rectum **(b)** volumes over 20 sessions and planning CT. Dotted line connects the threshold values—the middle of the rectum volume range for each patient

As mentioned above, the hypothesis is that a full bladder will improve bladder dose sparing, which we aim to prove quantitatively in this study. Although patients are asked to follow the standardized drinking protocol^1^, the bladder filling during the daily CBCT still has a wide distribution (see Fig. 1a and Fig. 1 in the Supplement), allowing for analysis of the correlation between the bladder volume and the dose-volume metrics. We focus on the clinically relevant dose-volume metrics V60Gy, V48Gy and V40Gy, where VxGy refers to the relative volume of the organ receiving xGy or more. For all metrics, the correlation with bladder volume is assessed using the Pearson correlation coefficient. Significance of difference between the bladder volume during planning CT and the bladder volumes during the sessions for each patient was obtained with the Wilcoxon one-sample test.

For the rectum, the analysis is less straightforward, since any variations in craniocaudal extent of the contouring, which may arise in the adaptation process, strongly influence the calculated organ volume. Furthermore, the doses calculated for the posterior rectal wall are very sensitive even to smallest changes in the anterior edge of the contour. To reduce variations in anterior contour edge, e.g. by different oncologists performing the adaptation process in different treatment fractions, in the clinical routine a semi-automated algorithm was introduced for contouring the PRW rather than manual adaptation on each treatment day. Thereby, the PRW is now created in a number of steps: First, the adapted rectum contour is expanded by 0.2 cm in the anterior-posterior direction and laterally by 5 cm. The aim of this is to even out the edges and create a smooth, linear anterior border to the PRW. The resulting help structure is then shrunk by 1.3 cm in the posterior direction, thus eliminating the posterior 1.3 cm of the rectum. The freely selected parameter of 1.3 cm is an empirical value that is appropriate for all regarded clinical cases. The original rectum is then cropped by this second structure, so that only parts contained within the rectum (posterior part) is retained (Fig. 2 in the Supplement). This procedure is defined as an automated rule in the Ethos system, so the daily adapted PRW contour will automatically be created from the propagated rectum contour. For the analysis, we hence select those 9 patients for whom this algorithm was implemented to provide more self-consistency.

Since we aim to compare doses between full and voided rectum, patients with low variability of rectum volume over all 20 sessions were excluded from the analysis. Whether the variation in filling was insufficient was defined by the following criterion: standard deviation of the rectum volume $\sigma_{\mathrm{Vrect}}<10\,\mathrm{cm}^{3}$. Patient #8 and patient #10 with $\sigma_{\mathrm{Vrect}}5.1\,\mathrm{cm}^{3}$ and $8.9\,\mathrm{cm}^{3}$ respectively were therefore excluded from the further analysis.

Several dose metrics are used in-house for the posterior rectum wall, namely V37Gy, V30Gy, and Dmax (see Table 1). Dmax is the maximum dose received by any part of the organ. It reflects very small areas receiving a given dose and highly depends on contouring quality/accuracy. Thus, we deem it unsuitable for estimating the dose variations to the PRW. We therefore concentrate the analysis on V30Gy, which is a parameter of intermediate dose with sufficient changes between the fractions to reflect changes in PRW sparing. V37Gy is also taken into account as a complementary parameter. The rationale for its secondary role is twofold: firstly, V37Gy, like Dmax, is significantly influenced by the contouring quality/accuracy, secondly, it is predominantly either small ($\mathrm{V37Gy}<1\%$ in 33% of sessions in the analysis) or equal to zero (in 48% of sessions) and, rendering it less informative for our analysis.

Since the variations in rectum filling are less pronounced in the considered collective of patients as the variation in bladder filling, and more sessions were applied with an empty rather than with a full rectum, we opted to dichotomize rectal filling as either “empty” or “full” instead of calculating correlation coefficients. This classification was determined by dividing the range of rectum volumes into half, selecting $V_{\mathrm{thr}}=(V_{\mathrm{min}}+V_{\mathrm{max}})/2$ as a threshold volume for each patient. For seven out of nine patients, this threshold exceeded both the median and mean rectum volume (see Fig. 1b), as the patients predominantly arrived with an empty rectum according to the recommendation. To assess the statistically significance of difference in dose for empty versus full rectum, the Wilcoxon rank-sum test (Mann-Whitney U Test) was conducted for each patient individually. Furthermore, for the entire patient collective, the paired Wilcoxon test was employed to compare the average values (empty versus full).

All statistical analysis was performed in OriginPro V2019b. Due to the exploratory nature of the study, $p$-values are descriptive; a $p$-value of $<\!0.05$ was considered indicative of statistical significance.

## Results

### Bladder dose-volume dependence

An example of different bladder fillings and resulting dose distributions for two treatment sessions for the same patient is shown in Fig. 1 in the Supplement.

For all except one patient (patient #12), metrics V48Gy and V40Gy show significant inverse correlation with the bladder volume with correlation coefficient $r$ between $-0.5$ and $-0.9$ and $p<0.05$ (Fig. 3 in the Supplement). A closer examination of patient #12 reveals that one CBCT was of very low quality, which led to inconsistent contouring, unfortunately not recognized and corrected immediately during the treatment. Upon excluding this problematic scan, the correlation substantially strengthens (compare the bottom-left and the bottom-right panels in Fig. 3 in the Supplement and Table 3, values to the left and to the right of the arrows) with [$r_{\mathrm{V60}}$, $r_{\mathrm{V48}}$, $r_{\mathrm{V40}}$] changing from $[-0.37,-0.35,-0.35]$ to $[-0.48,-0.57,-0.54]$.

The metric V60Gy exhibits an inverse correlation with the bladder volume for all patients, although it is only significant for 7/14 patients.

**Table 3:** Correlation between bladder volume and the metrics V60Gy, V48Gy, V40Gy. Pearson correlation coefficients $r$ and $p$-values are provided for each patient and metric (with significant correlations indicated in **bold** type). When several original plans are available but based on the same CT, only the first one is included in estimating the mean values

Patient	Bladd., $\mathrm{cm}^{3}$		V60Gy, %		Corr.	V48Gy, %		Corr.	V40Gy, %		Corr.
	Mean		Mean		$r$	Mean		$r$	Mean		$r$
	min	max	min	max	$p$	min	max	$p$	min	max	$p$
1	216		3.6		$\boldsymbol{-}$ **0.74**	15.5		$\boldsymbol{-}$ **0.90**	20.9		$\boldsymbol{-}$ **0.90**
	108	628	0.6	7.4	$\boldsymbol{<} \textbf{0.001}$	5.7	21.5	$\boldsymbol{<} \textbf{0.001}$	8.2	28.2	$\boldsymbol{<} \textbf{0.001}$
2	192		3.3		$\boldsymbol{-}$ **0.53**	19.1		$\boldsymbol{-}$ **0.89**	25.0		$\boldsymbol{-}$ **0.91**
	91	469	1.2	5.7	**0.014**	9.3	25.7	$\boldsymbol{<} \textbf{0.001}$	12.4	33.2	$\boldsymbol{<} \textbf{0.001}$
3	262		1.1		$-$0.42	11.9		$\boldsymbol{-}$ **0.90**	17.0		$\boldsymbol{-}$ **0.91**
	74	507	0.5	1.9	0.060	5.1	21.1	$\boldsymbol{<} \textbf{0.001}$	7.4	30.0	$\boldsymbol{<} \textbf{0.001}$
4	194		1.9		$-$0.25	15.1		$\boldsymbol{-}$ **0.89**	20.6		$\boldsymbol{-}$ **0.92**
	80	334	0.1	4.1	0.27	8.9	23.2	$\boldsymbol{<} \textbf{0.001}$	12.5	30.7	$\boldsymbol{<} \textbf{0.001}$
5	236		1.0		$-$0.40	15.4		$\boldsymbol{-}$ **0.86**	21.4		$\boldsymbol{-}$ **0.87**
	110	516	0.2	3.6	0.074	8.3	24.7	$\boldsymbol{<} \textbf{0.001}$	11.8	33.1	$\boldsymbol{<} \textbf{0.001}$
6	191		2.1		$-$0.19	19.5		$\boldsymbol{-}$ **0.65**	25.9		$\boldsymbol{-}$ **0.70**
	69	481	0.4	4.4	0.42	9.3	39.9	**0.0015**	12.8	49.4	$\boldsymbol{<} \textbf{0.001}$
7	274		1.3		$-$0.16	10.7		$\boldsymbol{-}$ **0.91**	14.7		$\boldsymbol{-}$ **0.92**
	107	540	0.3	2.5	0.48	6.0	18.1	$\boldsymbol{<} \textbf{0.001}$	8.4	23.9	$\boldsymbol{<} \textbf{0.001}$
8	179		1.7		$-$0.34	14.6		$\boldsymbol{-}$ **0.81**	20.5		$\boldsymbol{-}$ **0.80**
	110	311	0.4	3.1	0.14	8.2	21.1	$\boldsymbol{<} \textbf{0.001}$	12.4	27.7	$\boldsymbol{<} \textbf{0.001}$
9	156		2.4		$\boldsymbol{-}$ **0.47**	13.3		$\boldsymbol{-}$ **0.70**	19.2		$\boldsymbol{-}$ **0.71**
	85	465	1.1	4.2	**0.031**	7.4	20.2	$\boldsymbol{<} \textbf{0.001}$	10.4	28.2	$\boldsymbol{<} \textbf{0.001}$
10	100		1.9		$-$0.12	19.9		$\boldsymbol{-}$ **0.53**	28.2		$\boldsymbol{-}$ **0.61**
	59	279	0.4	5.7	0.60	10.1	36.9	**0.013**	14.3	48.6	**0.0031**
11	126		2.8		$\boldsymbol{-}$ **0.72**	13.6		$\boldsymbol{-}$ **0.73**	19.0		$\boldsymbol{-}$ **0.66**
	69	181	1.4	5.6	$\boldsymbol{<} \textbf{0.001}$	10.6	18.3	$\boldsymbol{<} \textbf{0.001}$	14.9	23.6	**0.0011**
12	144		3.3$\rightarrow$		$-$0.37$\rightarrow$	12.2$\rightarrow$		$-$0.35$\rightarrow$	15.9$\rightarrow$		$-$0.35$\rightarrow$
			2.8		$\boldsymbol{-}$ **0.48**	10.8		$\boldsymbol{-}$ **0.57**	14.4		$\boldsymbol{-}$ **0.54**
	90	364	0.3	12.7$\rightarrow$	0.099$\rightarrow$	4.2	40.2$\rightarrow$	0.12$\rightarrow$	6.2	45.0$\rightarrow$	0.12$\rightarrow$
				6.8	**0.034**		18.6	**0.009**		22.4	**0.015**
13	335		0.7		$\boldsymbol{-}$ **0.61**	6.5		$\boldsymbol{-}$ **0.86**	9.1		$\boldsymbol{-}$ **0.88**
	158	550	0.4	1.6	**0.0034**	3.7	15.2	$\boldsymbol{<} \textbf{0.001}$	5.0	20.0	$\boldsymbol{<} \textbf{0.001}$
14	330		1.9		$\boldsymbol{-}$ **0.67**	11.8		$\boldsymbol{-}$ **0.81**	16.6		$\boldsymbol{-}$ **0.82**
	159	521	1.0	4.0	$\boldsymbol{<} \textbf{0.001}$	6.6	19.8	$\boldsymbol{<} \textbf{0.001}$	9.6	28.0	$\boldsymbol{<} \textbf{0.001}$

**Fig. 2:**
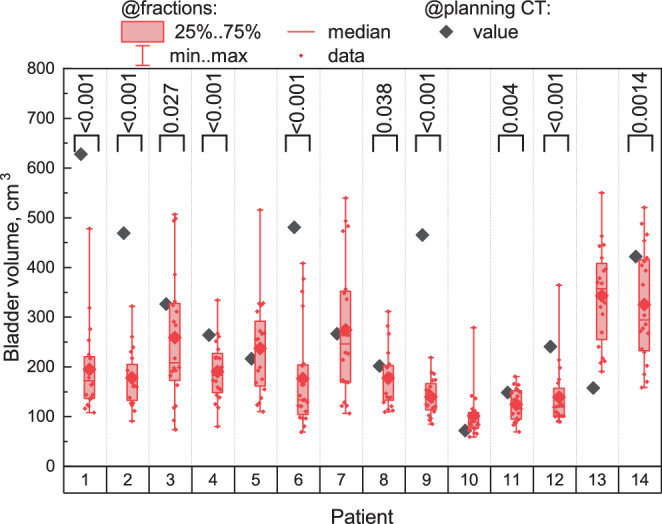
Red boxplots depict the bladder volume distribution over 20 sessions for each patient individually. A single grey point for each patient indicates the bladder volume on the planning CT. The brackets indicate cases where the bladder volume on the planning CT is significantly higher than the median over 20 sessions, with corresponding $p$-values are provided on top

**Fig. 3:**
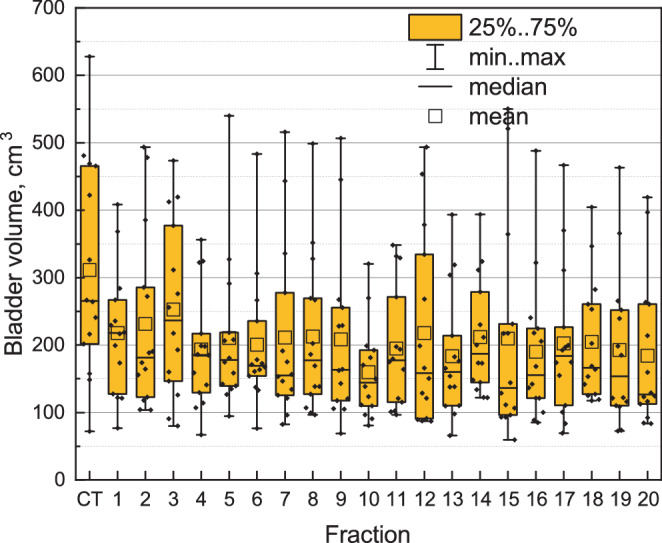
Trend in bladder volume. Each box represents data for the entire patient cohort for a single fraction or planning CT

We analysed the bladder volume during the planning CT, $V_{\mathrm{CT}}$, and the bladder volumes during the treatment sessions (see Fig. 2). $V_{\mathrm{CT}}$ is significantly higher than the median bladder volume over 20 fraction for 10/14 patients (based on the Wilcoxon one-sample test with the null hypothesis $\mathrm{H}_{0}$: $V_{\mathrm{CT}}\leq V_{\mathrm{med}}$). For 2/14 patients (##10, 13), $V_{\mathrm{CT}}$ is significantly lower than the median over 20 sessions (with $p$-values being $<0.001$ according to the Wilcoxon one-sample test with the null hypothesis $\mathrm{H}_{0}$: $V_{\mathrm{CT}}\geq V_{\mathrm{med}}$). For the remaining 2/14 patients (##5, 7), there is no significant difference between them. To ascertain whether a consistent trend in the bladder filling exists, we conducted the Wilcoxon signed-rank test for paired data between pairs of adjacent sessions and additionally between the first and the last sessions. No significant difference was observed, except for 2 cases: the bladder volume on the 3rd session was significantly higher than on the 4th session with ${p}={0.025}$ (the volume decreased), and on the 10th session, it was significantly lower than on the 11th session with ${p}={0.010}$ (the volume increased). Given the number of pairwise comparisons (and the omission of a Bonferroni correction), these two exceptions can be coincidences and are not sufficient to show any trend. However, the bladder volume was consistently higher on the planning CT than on any session starting from the first one as shown in Fig. 3 (being significantly higher in comparison with 13/20 sessions according to the Wilcoxon signed-rank test for paired data with $\mathrm{H}_{0}$: $V_{\mathrm{CT}}\leq V_{i}$, where ${i}$ is a session number).

### Rectum dose-volume dependence

**Fig. 4:**
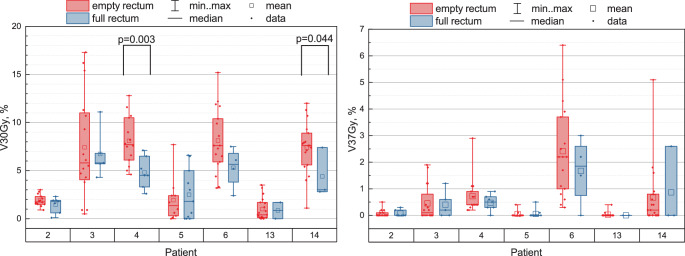
V30Gy **(a)** and V37Gy **(b)** for the PRW under empty (*red left boxes* for each patient) and full (*blue right boxes*) rectum condition. Each group of two boxplots represents data for one patient. Brackets indicate cases where V30Gy is significantly higher for the empty rectum condition than for the full one with $p$-values provided on top of the brackets

We compared V30Gy for the posterior rectal wall under two rectum conditions: full and empty (see Fig. 4a). To evaluate the significance of the difference in dose levels between these conditions, we conducted the Wilcoxon rank-sum test for unpaired data, also known as the Mann-Whitney U test, with the null hypothesis $\mathrm{H}_{0}$: $\mathrm{V30Gy}_{\mathrm{empty}}\leq\mathrm{V30Gy}_{\mathrm{full}}$. The brackets in Fig. 4 indicate statistically significant differences with $p$-value provided on top. For patients ##4, 14, V30Gy in the empty rectum condition was significantly higher than in the full rectum one, with $p$-values of 0.003 and 0.044, respectively. However, for 5/7 patients (##2, 3, 5, 6, 13), the $p$-values were $> 0.05$, indicating that the full rectum condition did not significantly improve V30Gy.

**Fig. 5:**
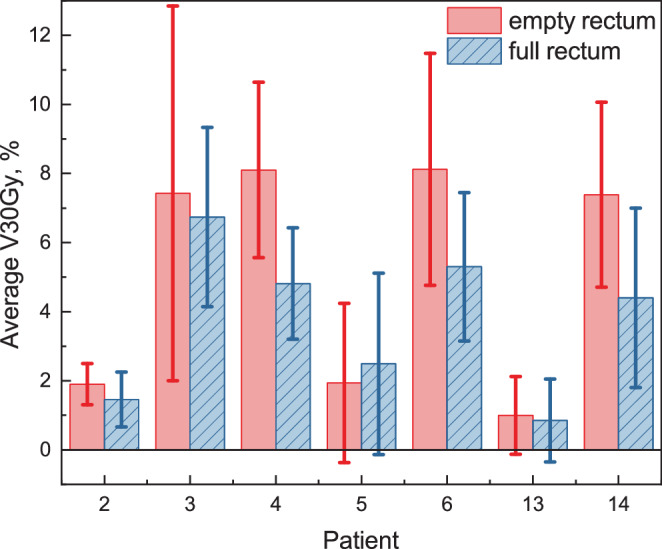
Average V30Gy for the PRW for each patient over 20 sessions and one planning CT with data presented for empty (red) and full (blue, hatched) rectum conditions. Error bars represent standard deviation

The analysis of the V37Gy showed no significant difference between full and empty rectum conditions.

**Table 4:** V30Gy metric for each patient: comparison between empty and full rectum conditions. Mean values are presented along with standard deviations

Patient	V30Gy, %		
	Empty rectum	Full rectum	Empty–full
	mean $\pm$ std.dev.		
2	$1.9\pm 0.6$	$1.5\pm 0.8$	0.4
3	$7.4\pm 5.4$	$6.7\pm 2.6$	0.7
4	$8.1\pm 2.5$	$4.8\pm 1.6$	3.3
5	$1.9\pm 2.3$	$2.5\pm 2.6$	$-$0.6
6	$8.1\pm 3.4$	$5.3\pm 2.2$	2.8
13	$1.0\pm 1.1$	$0.9\pm 1.2$	0.1
14	$7.4\pm 2.7$	$4.4\pm 2.6$	3.0

We averaged the V30Gy for each patient and each rectum condition (empty vs. full) separately. Considering the entire patient cohort, the Wilcoxon signed-rank test for the average values (empty vs. full) yielded a $p$-value of 0.039, indicating that the average V30Gy in the empty rectum condition was significantly higher than in the full one (Fig. 5, Table 4).

## Discussion

### Bladder

The study confirmed the expected validity of the standard recommendation to maintain a full bladder during the treatment sessions. We observed significant inverse correlation between V48Gy, V40Gy and the bladder volume. Additionally, V60Gy metric exhibited significant inverse correlation for 7/14 patients. One possible explanation for only half of the patient cohort showing significant inverse correlation is the low values of V60Gy, leading to a stronger influence of contouring onto V60Gy values. Smith et al. [17] confirmed that optimal bladder dose constraints missed only when the bladder volume was $<200\,\mathrm{cm}^{3}$. However, the bladder volume tended to increase while waiting for treatment after acquiring session images for plan adaptation, reducing the number of unmet goals.

Another observation was made regarding bladder volume on the planning CT and during treatment sessions. The patients tended to have higher bladder volume during the planning CT compared to the median volume over the sessions. This trend was particularly evident in patients ##1, 2, 6, 9 (see Fig. 2 and Fig. 4 in the Supplement: 10/14 patient showed a significant difference between bladder volume during the planning CT and the median bladder volumes over the sessions). We suppose that there may be several possible reasons for this: known treatment side effects include cystitis, and increased frequency of passing urine. Even when moderate, this may make it harder for the patients to maintain an adequate bladder filling, since they may feel more urge, increasing their apprehension that leakage may occur during treatment. The more prominent these effects become, the smaller may the bladder volume be, causing increased dose to the bladder and hence contributing to increasing the side effects.

**Fig. 6:**
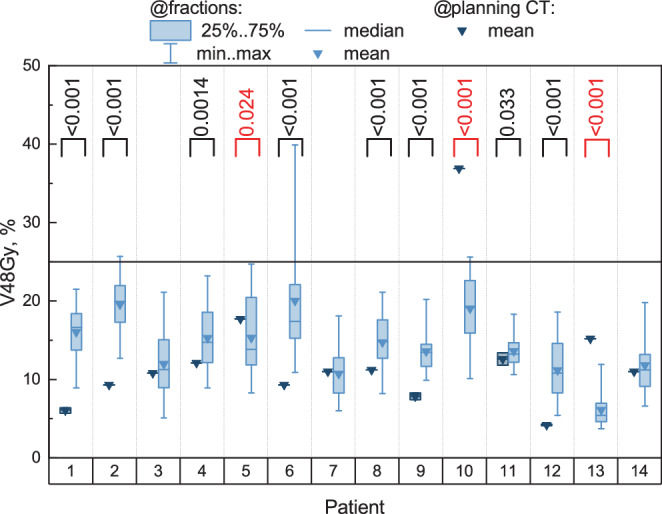
Blue boxplots present V48Gy for the bladder over 20 sessions for each patient individually. Dark-blue single points indicate V48Gy for the original plan (based on the planning CT). For some patients (## 1, 9, 11, 12), there are multiple original plans based on the same planning CT with slightly different values for V48Gy, thus these values are also presented as boxplots. The black horizontal line displays our in-house constrain for V48Gy: 25%. Black brackets indicate cases where the median V48Gy over 20 sessions is significantly higher than V48Gy from the original plan (or its mean value over several original plans if present), with $p$-values provided on top. Red brackets indicate the opposite cases, where the median over 20 sessions is significantly lower than in the original plan. Significance was obtained from the Wilcoxon signed-rank test for one sample

Some authors have claimed that bladder filling gradually decreases with each treatment session. Byun et al. [18] demonstrated a significant decrease in bladder volume over 5 fraction (the entire treatment in that case) despite their full bladder protocol. Grün et al. [19] also observed a decreasing trend in the bladder volume during therapy (40 sessions), although they utilized a drinking protocol with biofeedback, potentially influencing the bladder filling. Conversely, other authors [20, 21] did not observed any significant difference in bladder volume between planning CTs and CBCTs.

We also do not confirm the gradual volume decrease for the analyzed cohort of patients. The bladder volume was higher on the planning CT than on each session starting directly from the first one for the entire patient cohort (Fig. 3). However, no gradual decrease from session to session was evident.

Thus, the aforementioned side effects of radiotherapy cannot explain insufficient bladder filling from the first radiotherapy session onwards. There may be a psychological component in that the patients expect side effects and are possibly more cautious about presenting with a full bladder for the planning CT than for the treatment sessions due to the increased stress and exhaustion during daily treatment.

Based on our data, we can conclude at most a vague tendency for the bladder filling to be rather larger at the time of the planning CT than in the treatment sessions. However, if this should hold true, we might consider in the plan creation that even with adaptive treatment, the optimal bladder sparing from the original plan may not be maintained in reality (see Fig. 6). Therefore, in the original plan creation, it might be worthwhile to optimize bladder sparing beyond the threshold normally deemed acceptable, to leave “room for deterioration” in the treatment sessions with predominantly lower bladder filling. Adaptive treatment can only mitigate the shortcoming of insufficient bladder sparing to some degree; still the advantage of daily reoptimizing the plan will be to push bladder sparing even harder because it will try to hold the pre-established objectives even for smaller bladder volumes. This should be advantageous to the degree this can be achieved. Still, the most important contribution to good bladder sparing remains organ filling, so even with adaptive therapy the patients should be reminded of the importance of adhering to the drinking protocol and not voiding the bladder shortly before treatment.

### Rectum

Our study demonstrated that the PRW dose (represented by the V30Gy metric) was either significantly higher or at least not lower with an empty rectum compared to a full one. Notably, none of the patients experienced worse PRW sparing with the full rectum condition. This suggests that the full rectum condition does not compromise the quality of the achievable dose distribution, allowing us to reconsider the recommendation of the rectum filling.

Byun et al. [18], in their analysis of prostate stereotactic body radiation therapy (non-adaptive), observed a decreasing trend in rectum volume over the fractions (for the entire patient cohort). They noted a small but significant increase in mean dose to the rectal wall and a small but significant decrease in D2cc (maximum doses to $2\,\mathrm{cm}^{3}$), with no correlation addressed between rectum volume and dose. However, the authors concluded that these relatively small changes in dose did not correlate with any detrimental clinical outcomes.

Lebesque et al. [22] found no correlation between rectum filling and the high-dose rectum wall volume for conformal radiotherapy when analyzing all patients together. They suggested that increased rectum filling results in the outer rectum contour expanding both inside and outside the high-dose region. While the authors did not explicitly discuss the PRW, it can be assumed that the PRW expands outside the high-dose region (and the anterior wall—inside this region) receiving a lower dose with higher rectum filling. This conclusion is consistent with our results.

A question to be considered in this context may be, however, to what extent rectal filling will influence rectum (and prostate) mobility. It is well known that the prostate moves during the treatment fraction on a scale of ca. 5 mm [23]. Hypothetically, intra-fractional rectum movement might be higher with a full rectum compared to an empty one, which would be a concern if adaptation and/or treatment are too slow in comparison with rectal movement. Alexander et al. in [24] addressed this question, finding that larger rectal volumes did not predict greater intra-fraction prostate motion. They do not support the need for rectal preparation when delivering contemporary image-guided radiotherapy to the prostate.

Our findings align with expectations, as a full rectum can be considered an analogue to an endorectal balloon (ERB), which used to be applied to distance the posterior rectal wall from the high-dose region. Several studies confirmed the dosimetric advantage of ERBs or other types of spacers [8, 25–27]. Moreover, ERB usage significantly reduced intra-fractional prostate motion [28]. A problem with the balloon—in addition to patient discomfort—was poor reproducibility in positioning, which led to the abandoning of this technique in most institutions, giving way to the “empty rectum” paradigm for good reproducibility. With the onset of daily adaptive radiotherapy, it is time to reconsider some of these paradigms and question which of them remain clinically useful and beneficial. Rectal filling appears to be advantageous over an empty rectum as long as online adaptation ensures adequate dosimetric coverage of the target volume and sparing of organs at risk.

## Strengths and Limitations

This study has several strengths and limitations.

Since the research question was not considered during the treatment of the patients, there was no potential for conscious bias in the contouring and/or planning. This strengthens the validity of the findings, as the contours were not influenced by any preconceptions related to the study’s objectives.

While the post-hoc nature of the analysis is a strength, it also presents a limitation. The post-hoc analysis may introduce bias, as researchers might unintentionally focus on interesting patterns that arise from the data, which can lead to false positives.

Although our institution adheres to the QUANTEC recommendations [10], we cannot guarantee that these guidelines were strictly followed during adaptive radiotherapy. Time constraints during contouring, performed while the patient is on the treatment table, may have affected adherence to these recommendations.

Another limitation is the small patient cohort. The study included only 280/180 fractions (for the bladder/rectum analysis), which limits the generalizability of the results. Furthermore, since this was not a prospective, randomized and blinded clinical trial, we cannot conclude that relaxing the rectal filling prescription will actually result in reduced rectal toxicity. Future studies with larger cohorts are necessary to reinforce our findings.

## Conclusion

We quantitatively demonstrated that bladder filling plays a crucial role in its optimal sparing. Therefore, even with the implementation of adaptive therapy, patients should be reminded of the importance of adhering to the drinking protocol and avoiding voiding the bladder shortly before treatment.

Conversely, the recommendation for rectum filling can be relaxed. Reproducibility of rectum filling is not critical for adaptive radiotherapy, and a filled rectum appears even to be advantageous over an empty one, provided that online adaptation ensures adequate dosimetric coverage of the target volume and spares the organs at risk. Thus, patients may be encouraged not to void their bowels shortly before treatment, as long as this is tolerated throughout the session. Adopting a more relaxed approach regarding rectum emptying could enhance patient compliance and comfort without compromising treatment efficacy.

## Caption Electronic Supplementary Material

Supplementary Materials

## Footnotes

1 Our in-house drinking protocol: 1. void the bladder and the bowel; 2. drink 350 ml liquid within 10 minutes; 3. move for one hour (a walk or light activities); 4. come to the appointment (a planning CT or a CBCT).
