# Correction to: Full bladder, empty rectum? Revisiting a paradigm in the era of adaptive radiotherapy

**DOI:** 10.1007/s00066-025-02459-z

**Published:** 2025-08-25

**Authors:** Hanna Malygina, Hendrik Auerbach, Frank Nuesken, Jan Palm, Markus Hecht, Yvonne Dzierma

**Affiliations:** Department of Radiotherapy and Radiation Oncology, Saarland University Medical Centre, Kirrberger Str. 100, 66421 Homburg, Saar Germany

**Correction to:**

**Strahlenther Onkol 2024**

10.1007/s00066-024-02306-7

The article “Full bladder, empty rectum? Revisiting a paradigm in the era of adaptive radiotherapy”, written by Hanna Malygina, Hendrik Auerbach, Frank Nuesken, Jan Palm, Markus Hecht and Yvonne Dzierma, was originally published under exclusive license to Springer-Verlag GmbH Germany, part of Springer Nature. As a result of the subsequent decision to publish the article under the open access model, the article’s copyright notice was changed on 04 August 2025 to © The Author(s) 2025 and the article is now distributed under a Creative Commons Attribution 4.0 International License, which permits use, sharing, adaptation, distribution and reproduction in any medium or format, as long as you give appropriate credit to the original author(s) and the source, provide a link to the Creative Commons licence, and indicate if changes were made.

The images or other third party material in this article are included in the article’s Creative Commons licence, unless indicated otherwise in a credit line to the material. If material is not included in the article’s Creative Commons licence and your intended use is not permitted by statutory regulation or exceeds the permitted use, you will need to obtain permission directly from the copyright holder.

To view a copy of this licence, visit http://creativecommons.org/licenses/by/4.0.

Open Access funded by: Open Access funding enabled and organized by Projekt DEAL

