# Supplementary material for: Full bladder, empty rectum? Revisiting a paradigm in the era of adaptive radiotherapy
Source: Strahlenther Onkol. 2024 Oct 29;201(1):47–56. doi: 10.1007/s00066-024-02306-7 (PMC12364992; doi:10.1007/s00066-024-02306-7)
Supplement: Supplementary file 1 — Supplementary Materials [file 66_2024_2306_MOESM1_ESM.pdf]

Full bladder, empty rectum? Revisiting a paradigm in the era of adaptive radiotherapy

1 Supplementary Materials

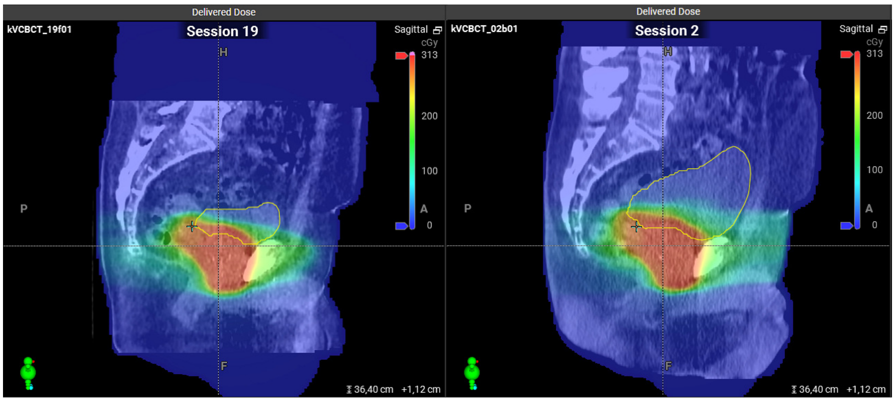

Supplementary Figure 1 A typical example of the dose distribution in the bladder for the same patient on different treatment days (session 19 on the left vs. session 2 on the right). Left panel: the bladder volume is 108 cm^3 (the lowest volume for the patient) and V60Gy = 5.2%, V48Gy = 18.8%, V40Gy = 24.4%. Right panel: 478 cm^3 (the highest volume for the patient) and V60Gy = 1.6%, V48Gy = 8.9%, V40Gy = 12.4%, correspondingly

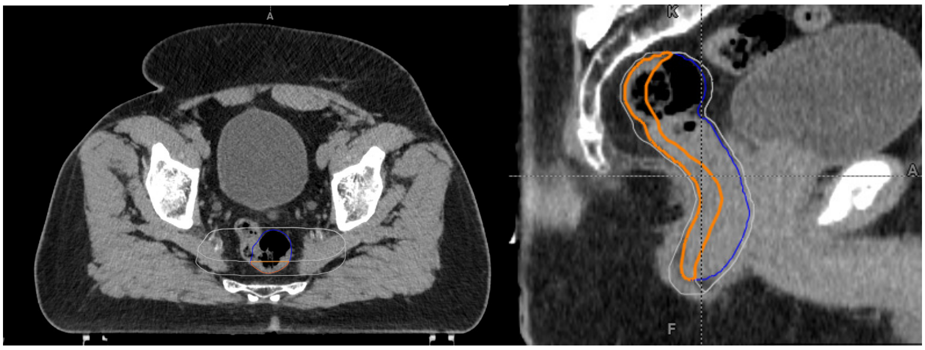

Supplementary Figure 2 Visualization of the help structures (white) defined to create the posterior rectum wall (PRW) contour (orange) automatically from the rectum contour (blue). This geometric algorithm aims to standardize the PRW delineation, minimizing inter-physician variability only in the anterior edge of the PRW contour. The reason for this is that on the one hand, the adaptation of the rectal contour is necessary to reproduce the anatomical variations. The rectal contour is adapted automatically and only edited by the physician if the anatomy is not delineated correctly. As such, the rectal contour is a very reliable structure which does not vary very much among the different physicians. However, the PRW has one great source of uncertainty, which is the decision where exactly to crop the anterior part. Since there is a significant dose gradient in this region, even a very small deviation in the anterior edge of this structure will greatly influence the DVH metrics. Furthermore, the AI which propagates the structures has no image information to guide the adapted delineation of the posterior rectal wall. While the AI will identify the rectum, the PRW can only be automatically delineated if defined by a specified algorithm based on the rectum contour. The algorithm we present solves this problem, so the PRW can be automatically created in a realistic and reproducible fashion.

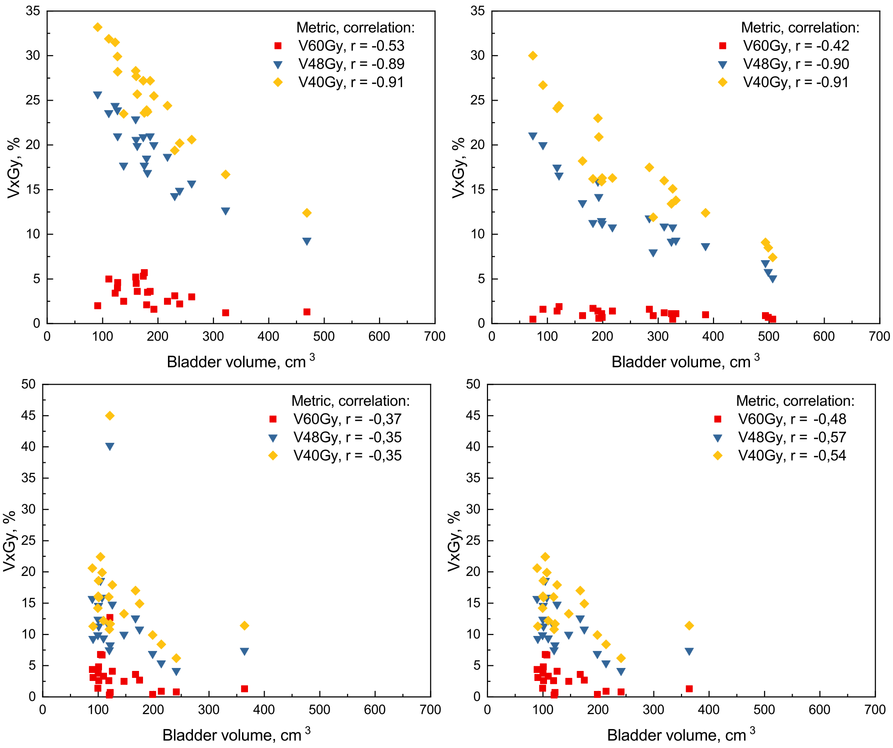

Supplementary Figure 3 Examples of correlation between the bladder volume and the metrics V60Gy (red squares), V48Gy (blue triangles), and V40Gy (yellow diamonds). The two top panels illustrate data for individual patients (#2 and #3); the two bottom panels represent data for patient #12: all fractions (left) and after exclusion of one single fraction with very low-quality CBCT and inconsistent contouring (right). Each point represents one fraction, and data from the planning CT is included as a single point. Correlation coefficients for each metric are provided in the legends

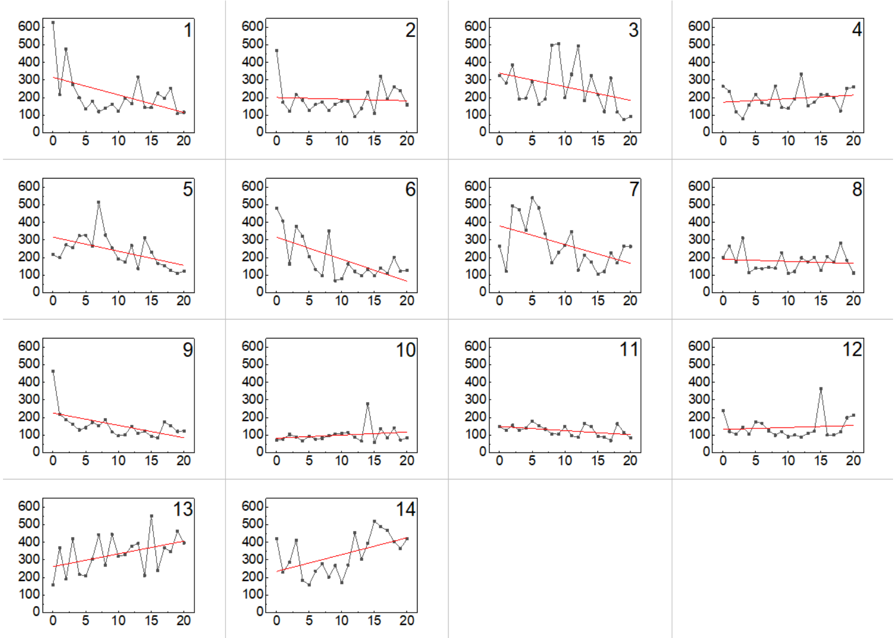

Supplementary Figure 4 Linear fits (red lines) of bladder volumes for each patient separately over 20 sessions, the planning CT corresponds to session #0. Each plot displays data for a single patient. The session number on the x-axis and bladder volume in cm^3 on the y-axis
